# Identifying the Turning Point: Using the Transtheoretical Model of Change to Map Intimate Partner Violence Disclosure in Emergency Department Settings

**DOI:** 10.5402/2012/239468

**Published:** 2012-06-26

**Authors:** Cristina Catallo, Susan M. Jack, Donna Ciliska, Harriet L. MacMillan

**Affiliations:** ^1^Daphne Cockwell School of Nursing, Ryerson University, POD 458B, Toronto, ON, Canada M5B 2K3; ^2^School of Nursing, Faculty of Health Sciences, McMaster University, Hamilton, ON, Canada L8S 4L8; ^3^Departments of Psychiatry and Behavioural Neurosciences and Paediatrics, Faculty of Health Sciences, McMaster University, Hamilton, ON, Canada L8S 4L8

## Abstract

*Background*. The transtheoretical model of change (TTM) was used as a framework to examine the steps that women took to disclose intimate partner violence (IPV) in urban emergency departments. *Methods*. Mapping methods portrayed the evolving nature of decisions that facilitated or inhibited disclosure. This paper is a secondary analysis of qualitative data from a mixed methods study that explored abused women's decision making process about IPV disclosure. *Findings*. Change maps were created for 19 participants with movement from the precontemplation to the maintenance stages of the model. Disclosure often occurred after a significant “turning point event” combined with a series of smaller events over a period of time. The significant life event often involved a weighing of options where participants considered the perceived risks against the potential benefits of disclosure. *Conclusions*. Abused women experienced intrusion from the chaotic nature of the emergency department. IPV disclosure was perceived as a positive experience when participants trusted the health care provider and felt control over their decisions to disclose IPV. *Practice Implications*. Nurses can use these findings to gauge the readiness of women to disclose IPV in the emergency department setting.

## 1. Introduction

The emergency department may offer a “window of opportunity” for women to discuss their experiences of intimate partner violence (IPV) with nurses and other health care providers. Exposure to IPV impacts women's health leading to acute and long-term physical and mental impairment [1–4]. Lifetime prevalence of IPV reported in the emergency department ranged from 37% [5] to as high as 50% [6]. The emergency department is a health care setting where abused women often seek treatment and may disclose at higher rates than other health care settings [7–9]. Due to the frequency with which abused women seek health care, many health care settings continue to adopt IPV-screening initiatives, and health care providers are encouraged to ask about violence as part of their routine care of women [4, 10, 11].

While abused women may seek care in the emergency department, many travel through the system without being recognized as exposed to violence [12]. This is problematic in that nurses, who may be the first point of contact for abused women, may miss an important opportunity for assessment. This is compounded by the fact that emergency department nurses, like other health care providers, continue to face challenges in the detection and documentation of IPV [13–15]. Common barriers to discussing IPV cited by nurses and other health care providers included a lack of knowledge about IPV, a lack of time to respond to IPV-related issues and disclosures, a fear of offending patients, and a perception that violence is not a priority for their practice area [4, 16, 17].

A past negative communication experience with a nurse might prevent abused women from subsequently disclosing IPV when seeking emergency care [18, 19]. In a meta-synthesis of 25 qualitative studies Feder et al. [20] explored women's perceptions of appropriate responses by health care providers to IPV disclosure. Abused women seeking to disclose IPV to a health care provider valued nonjudgmental, compassionate, sensitive responses, and desired confidentiality [20]. Other primary studies found women exposed to IPV favoured similar provider characteristics including open, empathic communication and a nonthreatening clinical environment [18, 19, 21]. Understanding the steps leading to IPV disclosure can improve nurses and other health care providers' awareness and facilitate non-judgmental approaches to IPV detection and response.

Chang et al. [22] recognized a gap in how health care providers understand and interpret abused women's decisions and behaviours when seeking safety. Very little information exists regarding how nurses and other health care providers engage in conversation with abused women and how they discuss safety issues related to IPV [22]. In order to address this gap, Chang et al. [22] used an innovative mapping technique based on the transtheoretical model of change (TTM) [23] to understand how women with a current or past history of IPV made changes that improved safety. Maps were created for safety-seeking behaviours, and results revealed that women made changes gradually, and over long periods of time. A significant event often marked the moment when participants adopted a change that supported their increased safety. This event was identified by Chang et al. as a “turning point” serving as a catalyst for change [22]. These authors found that abused women took many steps towards seeking safety, and these steps were often influenced by other factors that were beyond their control.

### 1.1. Transtheoretical Model of Change

The Transtheoretical Model of Change (TTM) [23] has been used to explore the dynamic processes that individuals undertake when making behavioural changes through the following stages: precontemplation, contemplation, preparation, action, and maintenance. In precontemplation individuals lack awareness of their problems and do not intend to change their behaviour [23]. Precontemplators experience denial about their situations and respond defensively and with resistance to external pressure to change [24]. When describing women exposed to IPV in this stage, Zink et al. [25] found that the women did not view their partners as abusive and described their relationships as normal. During contemplation, Prochaska et al. described a stage where participants became aware of their problem and considered how to deal with that problem. However, participants were not ready to take steps to change the problem [23]. When considering IPV, women in the contemplation stage acknowledged IPV as a problem but were not ready to leave their relationship [25]. When we consider women exposed to IPV, making changes may be limited to what women themselves have control over, such as their decisions to disclose violence and adopt safety strategies. During this stage, women weighed the pros and cons of their situation and the potential actions that they could take to address their resolve perceived problems [23]. While there were no set time periods for participants to move from stage to stage, authors stated that participants could remain in the precontemplation and/or the contemplation stages for years [23].

 During the preparation stage, participants plan their action. In some cases this action may have already been attempted over the past year [23]. Participants at this stage have not yet established criteria for effective action but had undertaken a series of small actions [23]. Chang et al. [22] stated that abused women sometimes experienced a “turning point” event that significantly altered their thinking and attitudes regarding IPV making it impossible to return to the precontemplation stage. According to another author, women exposed to IPV defined the following significant life events that served as impetus for “action”: a violent encounter, financial independence, or concerns about children [26]. These events, including those beyond the women's control, led them to take definitive steps to resolve the problem at hand. Chang et al. [22] found that women created plans to change during the preparation stage. The most obvious behaviour changes occurred during the action stage where participants committed time and energy to modify their behaviours [23].

Participants in the maintenance stage continued changing their behaviour and attempted to prevent going backwards to a previous stage [23]. When considering women exposed to IPV, Brown [24] argued that relapsing into a previously attempted stage may not be as likely to occur in the maintenance stage as it might in the action stage, since sustaining ongoing change can be challenging. Women in the maintenance stage would include those who have left their abusive partners and have attempted to rebuild their lives without returning to their partners.

There is a growing body of evidence regarding the use of the TTM [23] applied to women who have experienced IPV [22, 24–27]. Early research that applied the TTM with IPV focused on women leaving their partners as the ultimate outcome of change [28]. However, it was recognized that leaving the abuser could lead to reduced safety, diminishing resources and perpetuating ongoing harassment from the perpetrator [29, 30]. Brown [24] recommended that the health care provider refrains from using the outcome of leaving the relationship as an indicator of change.

Previous research explored the process of change among women who were preparing to leave abusive relationships or who left abusive relationships [22, 24, 26, 27, 29, 30]. Using the TTM when the focus is on women choosing to leave an abusive relationship is problematic in that abused women may not have complete control over these complex circumstances. Because of the complexity of abusive relationships where the perpetrator exerts control over the situation and many decisions, this impacts an abused woman's autonomy to make changes. As a result, we consider the use of the TTM in relation to the decisions and changes that women themselves can control, despite remaining in an abusive relationship, such as decisions to seek health care, disclosure of IPV to a nurse, and undertaking other types of social support. Little is known about the decisions and changes that abused women make when preparing to disclose IPV in emergency department settings. Recognition of the change stage of abused woman can help nurses to plan appropriate support for IPV disclosure.

### 1.2. Study Aim

This paper used an adapted form of the mapping methods proposed by Chang et al. [22] to understand how women move towards IPV disclosure in emergency departments. The TTM was used to interpret the sequence of events leading to IPV disclosure for a series of key participants. This paper examines the decision making steps that women undertake towards a change facilitating or inhibiting IPV disclosure. Four change maps will be presented with a description of each “turning point” that was the catalyst for change. Discussion will include how this mapping method can help nurses to gather information about a women's readiness to disclose IPV so that appropriate assessment, and interventions can be offered.

## 2. Methods

### 2.1. Study Design

This paper is based on a secondary analysis of a sequential explanatory mixed methods study. The mixed methods study involved a quantitative subanalysis of data from a randomized, controlled trial (RCT) followed by a grounded theory phase [31]. The RCT examined the effectiveness of routine screening for IPV in health care settings compared with usual care in reducing violence and improving life quality; its methods are reported elsewhere [32]. The grounded theory phase of the mixed methods study sought to explain the quantitative results and identify a core process related to IPV disclosure among a sample of women who participated in the trial [31]. From the grounded theory study, women exposed to IPV undertook a process to minimize intrusion resulting from the disclosure of violence in the emergency department. The process had three phases: (a) deciding to seek care, (b) evaluating level of trust with the presenting health care providers, and (c) establishing personal readiness to disclose. Abused women identified intrusion at various points including entry into a chaotic emergency department with long wait times and limited privacy, exposure to assessments requiring repeated descriptions of abuse to health care providers, feeling pressure to comply with evidence collection for potential legal proceedings, and/or involving other services such as police and/or child protection services [31].

### 2.2. Sampling

 Sampling for this mapping exercise occurred across the 19 participants in the grounded theory phase. While methods for sampling and data collection for the grounded theory phase of the mixed methods study are described elsewhere [31], theoretical sampling of participants involved the unit of analysis of “IPV disclosure events.” Disclosure events were used in order to obtain conceptual density and a theory grounded in the data, despite a bounded sample of participants from the RCT. This unit of analysis enabled constant comparison of findings in order to achieve theoretical saturation based on the incidents, events, or situations related to disclosure of IPV in urban emergency departments. The 19 women described 113 individual IPV disclosure events involving emergency department health care providers, social service professionals, police, family members, and friends [31]. Change maps based on Chang et al.'s [22] methods were completed for all 19 participants.

### 2.3. Data Collection

 Data for the grounded theory study was collected from 19 participants recruited from three emergency departments already enrolled in a RCT in Ontario, Canada from May 2006 to December 2007. Data collection involved face-to-face, in-depth, and semistructured interviews describing IPV disclosure events and lasted 60–90 minutes. After an initial interview that focused on establishing trust and a description of abuse experiences, participants completed up to four repeat interviews in order to explore emerging themes and to saturate theoretical concepts. Permission was obtained to review participant demographic data that was collected as part of the RCT. Each participant received $25 per interview as an honorarium.

### 2.4. Data Analysis

NVIVO 7.0 was used to organize data and complete coding of transcripts. In order to assess how participants moved through the TTM stages, an adapted form of Chang et al.'s [22] mapping methods was used. These authors briefly reported use of a grounded theory approach to coding to identify themes as they emerged from the data without use of a code book; rather predetermined codes related to the TTM were created and applied to the transcripts. For this study, data were analyzed according to the stages of the TTM, including a second review of transcripts, after the development of the grounded theory that explained the processes used by women to disclose IPV to health care providers in urban emergency department settings. Secondary analysis involved creating a coding structure for each participant's data according to the TTM stages. During coding, behaviours, events, and potential facilitators and barriers to disclosure were coded for the TTM stages. Data were reviewed for any potential factors related to the event that were beyond the participant's control (e.g., partner losing a job) and these were coded with the corresponding event. Once data were coded according to the TTM, each stage was examined closely for key behaviours and turning points that supported change towards IPV disclosure in an emergency department. Because this sample of 19 participants described multiple disclosure events to various types of health care and social service providers, change maps described the emergency department disclosure that participants identified as most significant in facilitating change.

After completion of the coding process, Chang et al.'s [22] methods were followed to map the stages of change per participant. These authors created a change map with an *x*-axis-depicting time and a *y*-axis-depicting stages of the TTM. For this analysis, time was not plotted due to the fact that movement through each stage was individual and may have occurred over multiple time periods. Another adaptation to Chang et al.'s [22] method was to chronologically plot critical events or factors on the map. These events or factors influenced a participant's movement from one stage to the next. Unique to this analysis was the inclusion of a large arrow to identify and describe a “turning point” or significant life event provoking disclosure of IPV. This event represented an important change in a participant's movement through the TTM stages toward action. Also unique to this analysis was the inclusion of events when participants “weighed options” and evaluated their perceived risks before taking action. Change maps were created and discussed with a master's student with content expertise in IPV who served as a second coder for transcripts. Any differences of opinion were discussed until consensus was achieved.

### 2.5. Ethics

Both the RCT and the grounded theory studies were approved by the McMaster University/Hamilton Health Sciences Research Ethics Board, Hamilton, Ontario, Canada and site-specific ethics boards. Written informed consent was obtained prior to study enrolment. A formal safety protocol was followed and each participant received a resource support card with local area organizations (available from the authors).

## 3. Results

### 3.1. Stages of Change

 Change maps revealed four participants in the precontemplation stage, six participants in the contemplation stage, three participants in the preparation stage, three participants in the action phase, and three participants in the maintenance stage. The majority of women did not move in a linear fashion through the stages of the TTM with the exception of three participants who moved from the precontemplation to the maintenance stage and disclosed IPV in the emergency department in a 12-month period. Those participants in the maintenance stage would then go on to disclose IPV to other health and social services providers in the future. Participants who disclosed IPV described a significant “turning point” event that influenced their movement toward the action of IPV disclosure. A summary of demographic characteristics is provided in Table 1.

Among those participants in the precontemplation stage, IPV was not perceived as a problem that required immediate or short-term action. For this group of participants, emergency department care was sought for immediate injuries and concerns related to IPV and not disclosure. For the six participants in contemplation, they identified various sources of intrusion as potentially influencing their movement towards IPV disclosure. Intrusion by nurses and other health care providers included judgmental responses for remaining in an abusive relationship or not following through on recommendations and an emphasis on efficient processing of clients through the emergency department. These participants also described intrusion from other services like the involvement of child-protective services and police. Involvement of these services was seen to be long term and lead to potential apprehension of their children and subsequent punishment of their partners. For these participants, disclosing IPV in an emergency department setting required greater consideration and preparation.

**Figure 1:**
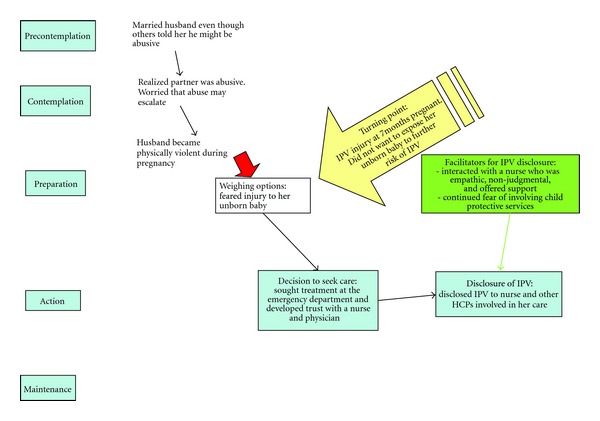
Example of a sequential movement through the stages of change.

**Figure 2:**
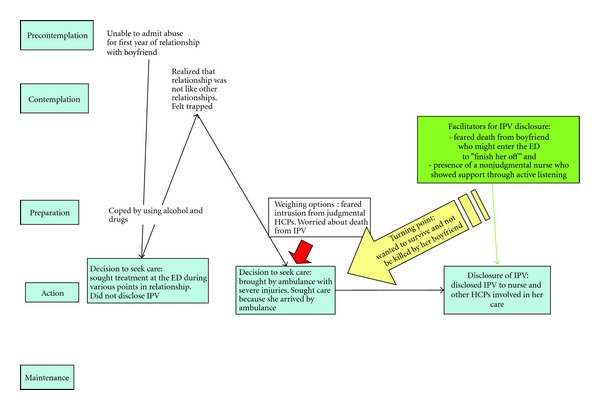
Example of a sequential movement through the stages of change.

 Figures 1 and 2 show two participants who moved through the TTM using a sequential process of change. Both carried out actions that they could control, such as a disclosure to an emergency department nurse.  Figure 1 shows a participant with the most linear movement through the TTM stages of change ending with a disclosure of IPV in the emergency department. This participant said that she had married her husband despite warnings from others that he had the potential to be verbally abusive. She sought care at the emergency department when she began to have frequent anxiety attacks, which she was able to relate to the ongoing verbal and emotional abuse from her husband. This participant did not identify her relationship as abusive and, upon realizing that it was, became embarrassed about her situation. The perceived risks associated with disclosure for this participant included experiencing judgment from health care providers, the fear of the violence “getting worse,” and fear that her husband would leave the marriage. Physical abuse did not begin for this woman until after she became pregnant; she sought care at the emergency department in order to protect her unborn child. For this participant, physical violence by her husband became more frequent as her first pregnancy developed. By the time the participant was seven months pregnant, a violent episode where she sustained physical injuries became her “turning point” to decide to seek care in the emergency department. While receiving treatment, the participant was able to identify a nurse whom she perceived as empathic and willing to listen to her story of abuse. For this participant, interaction with a non-judgmental and compassionate nurse was a facilitator to support her disclosure of IPV. Another facilitator that supported IPV disclosure for this participant was the fear that if she did not discuss IPV, her newborn would be apprehended at birth by Child Protective Services. This participant perceived disclosure to an emergency department nurse as a means of controlling when Child Protective Services would be involved in her life. 

Figure 2 depicts a participant who contemplated IPV disclosure and frequently sought emergency care. Over the course of many visits to the same emergency department, this participant prepared for IPV disclosure by assessing the trustworthiness of health care providers. A health care provider could be trusted if s/he was non-judgmental maintained confidentiality when discussing sensitive issues, despite these participant's frequent visits to the emergency department. The “turning point” event for this participant was severe physical injuries sustained from abuse from her partner. During her transport to the emergency department by ambulance, the participant considered the possibility of disclosure to a health care provider. One facilitating factor was her fear of death and survival through the night that became more important than the risks that she associated with disclosure. During this time, the participant also identified a nurse who provided support and a nonjudgmental approach. This care received from the nurse served as a facilitator for the participant who then disclosed IPV.

**Figure 3:**
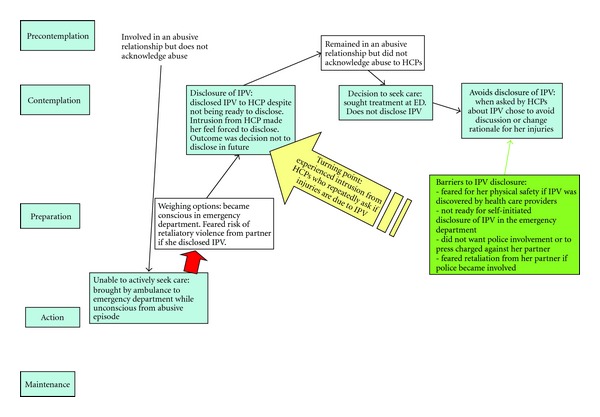
Example of a non-sequential movement through the stages of change.

For two participants, disclosure of IPV occurred prior to being ready to disclose, and these participants were in the preparation stage of the TTM. The maps for these participants show a partial, nonlinear, and nonsequential movement through the stages of change.  Figure 3 shows a change map for a participant who felt unable to control many decisions related to seeking health care and disclosure of IPV. For this participant, she felt forced to disclose IPV to a health care provider when brought to the emergency department by emergency services. Because the participant was unconscious at the time due to severe injuries, she struggled with the loss of control over her choices, such as when to seek health care. Her loss of control was further compounded by her perception of intrusion from health care providers within the emergency department who were providing her care. For this participant, her “turning point” arose when health care providers repeatedly asked her whether or not her injuries were due to IPV. The participant described feeling forced to disclose IPV to emergency department health care providers prior to a sense of readiness. While it appears that she took action by disclosing IPV, she remained in the contemplation stages of the TTM. This experience caused her to avoid disclosure on subsequent visits to the emergency department. Disclosing IPV, prior to being ready combined with perceived intrusion by health care providers, led to an avoidance of disclosure during future visits to the emergency department as a means of controlling her situation. Barriers to disclosure of IPV were the lack of readiness, the fear that disclosure would lead to police involvement, and the fear that her partner would retaliate if he discovered the disclosure or if police charged him with assault. Experiencing the “turning point” event of intrusion, this participant weighed her options and decided that the risks of retaliatory IPV from her partner outweighed her need to keep IPV under cover. Action taken by this participant reflected a nonsequential movement through the TTM and occurred as a result of the influence of others despite her desire to keep abuse a secret.

**Figure 4:**
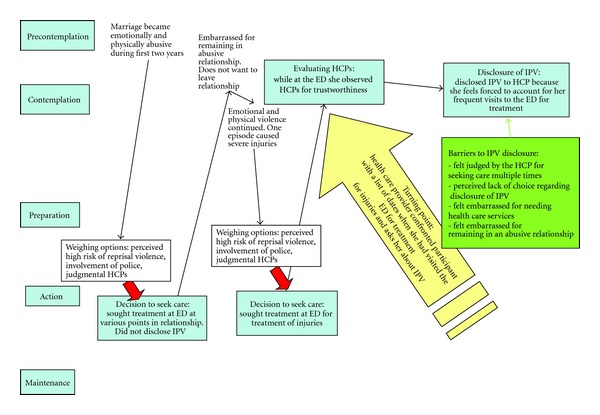
Example of a non-sequential movement through the stages of change.

Figure 4 shows a participant who also described a loss of control related to IPV disclosure in the emergency department. Similar to the previous participant, in Figure 4, this participant sought care in the emergency department on a regular basis for injuries sustained as a result of IPV. The “turning point” for this participant came when an emergency department health care provider approached her with a list of dates when she had sought care for her physical injuries. The health care provider then asked whether or not the participant sustained injuries from IPV. While she was still in the contemplation stages of the TTM, the participant disclosed IPV and felt forced to discuss her situation with the health care provider. Barriers to disclosure for this participant were experiencing judgement from the health care provider for seeking emergency care on multiple occasions, feeling shame for requiring care as a result of abuse, and feeling embarrassment for remaining in an abusive relationship. For the participants in Figures 3 and 4, neither felt a readiness to disclose IPV and felt forced to take the action of disclosing IPV prior to moving to the action stage of the TTM.

## 4. Discussion and Conclusion

 Chang et al.'s [22] methods for mapping provided an innovative way to explore abused women's movements through the stages of change. This mapping exercise gave a visual representation for the series of decisions and small actions toward IPV disclosure in the emergency department. Similar to the findings of Chang et al. [22], the majority of change maps for participants lacked linearity and included the multiple interruptions over time by external factors. These authors found that some participants skipped some of the stages of the TTM due to factors beyond their control like pregnancy, illness, and substance use. Likewise in our study, participants identified “turning point” events that served as catalysts for change. “Turning point” events served to initiate action, for example, self-initiated IPV disclosure. Some significant turning points that arose from this study were the fear of being killed by the perpetrator, fearing harm to an unborn baby or other children, needing emergency care for injuries related to violence, and feeling pressured to disclose IPV when asked by health care providers. This analysis provides new insights regarding participants who “weighed options” and considered their perceived risks of disclosing IPV with their perceived benefits. Despite a presentation of what appears to be a simplistic diagram of action steps, this exercise demonstrated that IPV disclosure in the emergency department is a complex process influenced by factors within and beyond the participant's control. For most of the women in this study, movement through the various stages of change took years and many visits to the emergency department that included treatment of injuries related to IPV. This exercise emphasized the role that clinicians can play in fostering client trust through communication and supporting a client's readiness for IPV disclosure.

 There are many important clinical implications that arise from this mapping exercise. Nurses who encounter abused women can consider the processes that women undertake when deciding to disclose IPV in the emergency department setting. Wong et al. [33] stated that health care providers undervalued the crucial role they can play in the response to women exposed to IPV. Among the participants of this study, IPV disclosure was found to be a positive experience when the nurse and other health care providers offered non-judgmental and empathic support. Considering how abused women move through the stages of the TTM can help guide nurse's conversations regarding IPV and work towards establishing the client's trust.

 This mapping exercise could be used in future research to identify appropriate interventions that best fit with the TTM stage of change. Several authors recommended patterning IPV-related intervention with the client's stage of change [24–26, 33]. For example, when a nurse might suspect that a client is exposed to IPV, she or he could include a discussion of the TTM and attempt to identify which stage of change is most applicable [26]. These authors stated that a woman's response to IPV-related questions indicated their stage within the TTM from denial of IPV (precontemplators) to describing strategies for how to deal with IPV (action). Authors recommended that nurses and other health care providers conduct “consciousness-raising” whereby the clinician educates abused women on the risks associated with IPV and expresses concern about their client's safety [25, 26, 33]. For the participants of this study, consciousness raising might have been helpful if it was provided over multiple occasions to the women who disclosed IPV. These women drew on any information or support from nurses and health care providers when they were actively preparing for disclosure.

 Among the women in earlier stages such as precontemplation and contemplation, however, this intervention by nurses could have been interpreted as invasive and created further feelings of shame among women seeking treatment in the emergency department. This was especially of concern among those participants who perceived their interactions with health care providers as invasive. These participants continued to be in a place of denial and perceived judgement for their decision to remain in an abusive relationship. Strategies to provide information about IPV in a noninvasive manner might assist participants concerned about further invasion from the health care provider.

 This mapping technique may help explain issues that challenge health care provider such as frequent and multiple visits to the emergency department by abused women [34]. These authors argued that nurses and health care providers frequently misunderstand client behaviours and become frustrated when women seek emergency care on multiple occasions. Women are often marginalized by the health care provider for the decisions they make in their relationships [34]. This lack of understanding of the strategies that women use to mitigate the risks that they associate with IPV disclosure could serve as a barrier to women attempting to establish the nurse's trustworthiness and, ultimately, their own internal readiness for IPV disclosure. This mapping activity can also support health care provider education by addressing the lack of education—or skills—barrier related to IPV frequently cited by participants [16, 17, 35, 36]. Use of change maps illustrates how taking action towards IPV disclosure is a long process involving multiple visits to the emergency department. Understanding this can help nurses to recognize the complexity of IPV disclosure, which is often a slow process towards change. Awareness of the factors that facilitate and impede IPV disclosure may help ease judgments against a client for her decision not to disclose IPV. In the absence of evidence for effective interventions related to IPV disclosure, the research provided by authors on stage-based approaches related to IPV can help shift nurses and other health care providers attitudes to women exposed to IPV from one of judgment to one of neutrality.

 One limitation of this study is its reliance on one set of informants—women exposed to IPV—to describe events and circumstances related to IPV disclosure. It would have been useful to include perspectives from nurses and other health care providers. It is difficult to discern whether participants shared all events related to the disclosure process or may have had difficulty recalling their experiences related to IPV disclosure. Future research could include women who had recently disclosed IPV within a specified time period, as opposed to participants who had disclosed over varied time periods.

 In conclusion, these findings suggest that few women followed a linear pattern using the Transtheoretical Model of Change. The mapping technique can be a useful for emergency department nurses working with abused women to begin and guide conversations related to IPV. Consideration of the complex process of change and conveying a willingness to discuss IPV may promote trust and engagement between client and nurse.

## Figures and Tables

**Table 1:** Demographic results for sample in grounded theory phase.

Demographic characteristic for sample (*N* = 19)	Sample mean
Age in years for total sample (*N* = 19)	30.7
Marital status for total sample	Married
Pregnancy status for total sample	Not pregnant
Number of children at home for total	1.2
Years of education for total	12.7
Main activity for total	Work full or part-time outside of the home
Main source of income for total	Wages or salary
Household income for total	Less than $24,000
